# Arsenate Removal from Aqueous Media Using Chitosan-Magnetite Hydrogel by Batch and Fixed-Bed Columns

**DOI:** 10.3390/gels8030186

**Published:** 2022-03-17

**Authors:** Ilse Paulina Verduzco-Navarro, Eduardo Mendizábal, José Antonio Rivera Mayorga, Maite Rentería-Urquiza, Alejandro Gonzalez-Alvarez, Nely Rios-Donato

**Affiliations:** 1 Chemistry Department, CUCEI, University of Guadalajara, Blvd. Gral. Marcelino García Barragán 1421, Guadalajara 44430, Jalisco, Mexico; pau.verduzco@yahoo.com (I.P.V.-N.); eduardo.mmijares@academicos.udg.mx (E.M.); jose.rivera@academicos.udg.mx (J.A.R.M.); maite.rurquiza@academicos.udg.mx (M.R.-U.); 2 Chemical Engineering Department, CUCEI, University of Guadalajara, Blvd. Gral. Marcelino García Barragán 1421, Guadalajara 44430, Jalisco, Mexico; alejandro.gonzalez0117@academicos.udg.mx

**Keywords:** adsorption, arsenic, chitosan, magnetite, hydrogel

## Abstract

The removal of arsenate ions from aqueous solutions at near-neutral pH was carried out using chitosan-magnetite (ChM) hydrogel beads in batch systems. Equilibrium isotherms and kinetic studies are reported. Obtained equilibrium and kinetic data were fitted to mathematical models, estimating model parameters by non-linear regression analysis. Langmuir model was found to best fit equilibrium data; a maximum adsorption capacity of 66.9 mg As/g was estimated at pH 7.0. Pseudo-first order kinetic model was observed to best fit kinetic data. The pH of the solution was observed to increase with increasing contact time, which is attributed to protonation of amine groups present in the hydrogel. Protonation of functional groups in the ChM sorbent yields a higher number of active sites for arsenate removal, being as this a process that can’t be overlooked in future applications of ChM hydrogel for the removal or arsenate ions. Chitosan-magnetite and ChM-arsenate interactions were determined by XPS. Arsenate removal using fixed-bed column packed with ChM was carried out, reporting a non-ideal behavior attributed to pH increase of the effluent caused by proton transfer to ChM hydrogels.

## 1. Introduction

High quality water availability is essential for the sustainment of life. Water pollution by hazardous materials is of particular interest due to the detrimental health effects related to its consumption. Arsenic is a pollutant that may originate from natural processes of sediment dissolution, but can also originate from anthropogenic sources derived from activities such as the burning of fossil fuels, mining and smelting, use of arsenic-containing agrochemicals, among others [1]. Arsenic present in water is mainly in the form of the inorganic compounds arsenite and arsenate [2,3], and under oxidizing conditions, the pentavalent form of arsenic is prevalent [2,4]. At near neutral pH, the predominant species of arsenate are the acid species HAsO_4_^2−^ and H_2_AsO_4_^−^ [5]. The prolonged ingestion of water with >10 µg As/L augments human mortality since it is capable of causing various cancer types [6]. Additionally, the ingestion of arsenic can be due to the consumption of arsenic-contaminated food, such as fish [7]. The toxicity of arsenic is also evidenced by its identification as a plant stressor [8,9].

The removal of pollutants from aqueous media can be carried out by the use of a diverse array of technologies, such as chemical coagulation, biological treatment, electrochemical oxidation, ozonation, ultrafiltration and adsorption [10]. Adsorption is widely used to remove contaminants due to the simplicity of its design and operation and low operating costs [11,12]. Adsorption may be described as the mass transfer process where a substance, in this case, the pollutant, is transferred to a solid surface due to physical chemical interactions [13,14].

Activated carbon is an effective universal adsorbent. However, its use in water treatment is restricted due to its high cost; a cost-effective alternative method for removing contaminants from water is the use of adsorbents obtained from agro-industrial wastes [15]. Chitin, obtained from seafood industry waste, is the second most abundant naturally occurring form of polymerized carbon [16,17]. Chitin can be partially deacylated for the obtention of chitosan, a biopolymer reported to be an effective adsorbent for the removal of ionic species, due to the presence of amine and hydroxyl groups in its structure capable of acting as active sites [18,19]. Chitosan is supplied in powder or flake form but is used as an adsorbent in the form of hydrogel beads [20,21,22] which is advantageous when used in large-scale water treatment with packed columns because the powder or flakes can cause a significant drop in hydrodynamic pressure or clogging of the column [20,23,24]. Improved functional properties of chitosan can be attained by combining it with other components to produce composites [25]. Chitosan can be utilized as a polymeric matrix to disperse other adsorbent materials or dispersed in other polymeric matrices (e.g., alginate), for the obtention of hydrogel-adsorbent composite beads.

It has been reported that chitosan beads have a maximum adsorption capacity at pH = 5.0 of 1.78 and 1.90 mg of As(III) and As(V), respectively, per gram of adsorbent. [26]. It was later reported that by impregnating chitosan with iron, the arsenic uptake capacity was significantly increased, reporting a capacity of 6.48 mg/g for As(III) when studying the adsorption process in batch systems at pH = 8.0 [23]. Iron oxides are considered to be useful as arsenic adsorbents since they form complexes with arsenate ions and are low-cost materials [27]; additionally, iron oxides present a high affinity for chitosan [6,28]. Chitosan-iron oxide composites have been used for the treatment of arsenic-contaminated groundwater, at pH of 7, using batch systems; adsorption capacities of 16.15 and 22.47 mg As/g adsorbent were reported for As(III) and As(V), respectively [29].

Chitosan-impregnated magnetic Fe_3_O_4_ nanoparticles have been studied for arsenic removal from aqueous systems, in a batch system at a pH of 6.8; maximum adsorption capacities of 35.3 and 25.7 mg As/g adsorbent were obtained for As(III) and As(V), respectively [30]. Arsenate was removed from the aqueous system using magnetite in a batch system at an initial pH of 7.9, where a maximum adsorption capacity of 3.65 mg As/g was obtained [6]. Magnetite is an iron oxide that has been an object of interest in water treatment research due to its biocompatibility, low-toxicity, magnetic property, low-cost, and its high affinity to arsenic species [31,32].

The present work shows the dispersion of magnetite nanoparticles in a chitosan hydrogel matrix to obtain hydrogel beads suitable for arsenate removal. Adsorption data were fitted to mathematical models, estimating model parameters by nonlinear regression analysis. Chitosan-magnetite and ChM-arsenate interactions were determined by XPS. Arsenate removal was also carried out in a fixed-bed column packed with the chitosan-magnetite hydrogel at near-neutral pH.

## 2. Results and Discussion

### 2.1. Characterization of ChM Hydrogel Beads

#### 2.1.1. Hydrogel Bead Composition

**Figure 1:**
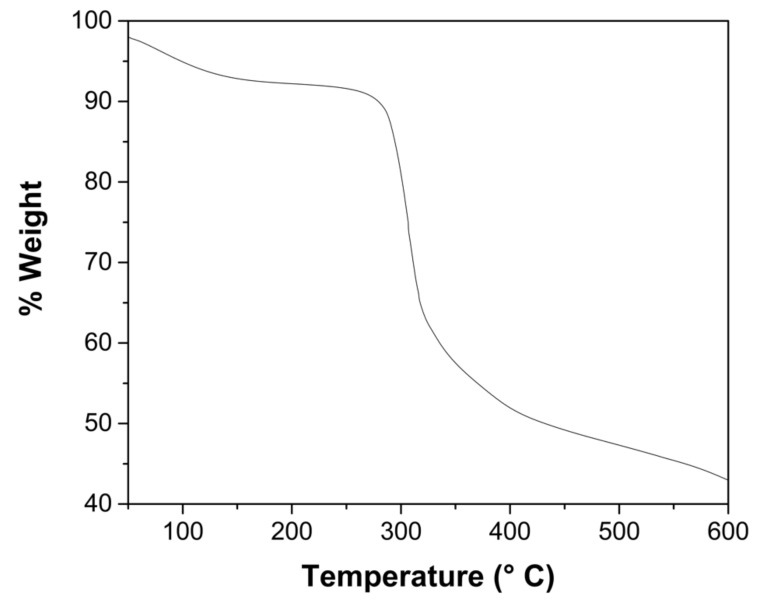
Thermogravimetric analysis of ChM powder.

It was obtained by gravimetry that the ChM contained 93.53 ± 0.10% water. Magnetite content of the dry beads was determined by thermogravimetric analysis (TGA). Figure 1 shows the thermal behavior of ChM, where 7.4% mass was lost within 160 °C, attributed to water loss, and 47.6% mass was lost in the 160–560 °C range, which is attributed to the decomposition of the chitosan matrix. From 560 to 600 °C, the decomposition process became slow and only 2.0% mass loss occurred. At 600 °C, 43.0% of the mass of the ChM residue remained. Thus, the magnetite content of the hydrogel ChM beads was of 2.73%, and the chitosan content of the hydrogel beads was of 3.74%.

#### 2.1.2. Average Diameter of the Hydrogel Beads

A sample of 60 hydrogel beads was measured, resulting in the average diameter of the ChM beads being 2.65 ± 0.07 mm.

#### 2.1.3. Potentiometric Titration of ChM

The pKa of ChM was obtained from the titration data and by its analysis by linear regression using the Henderson–Hasselbalch equation [33], obtaining a pK_a_ value of 6.19 ± 0.05.

#### 2.1.4. Morphology and Surface Characterization of Hydrogel Beads

**Figure 2:**
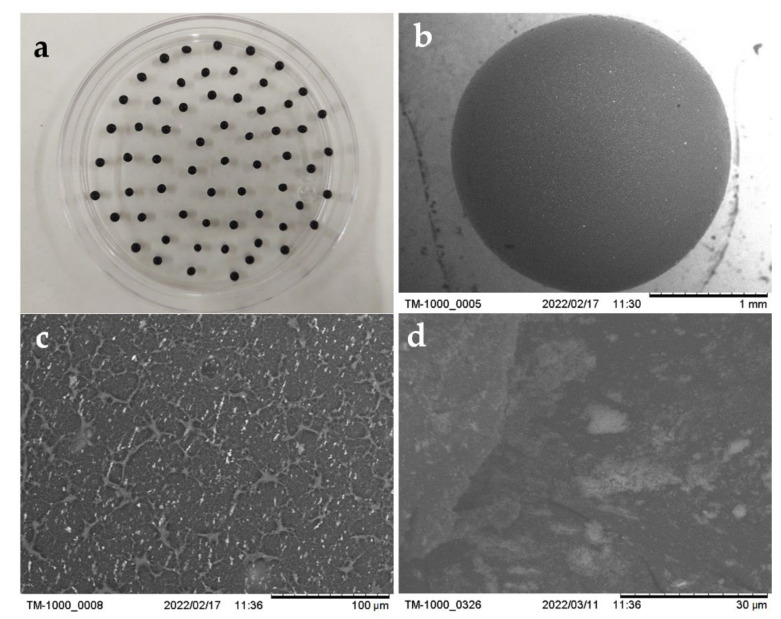
Images of ChM beads obtained (**a**) with a digital camera. (**b**–**d**) with a scanning electron microscope (SEM).

Figure 2 shows that the ChM hydrogel beads are spherical (Figure 2a,b) with an irregular surface with pores (Figure 2c). It can be observed in Figure 2d that some magnetite nanoparticles form clusters.

#### 2.1.5. Characterization of Chitosan-Magnetite and ChM-Arsenic Interactions by XPS

**Figure 3:**
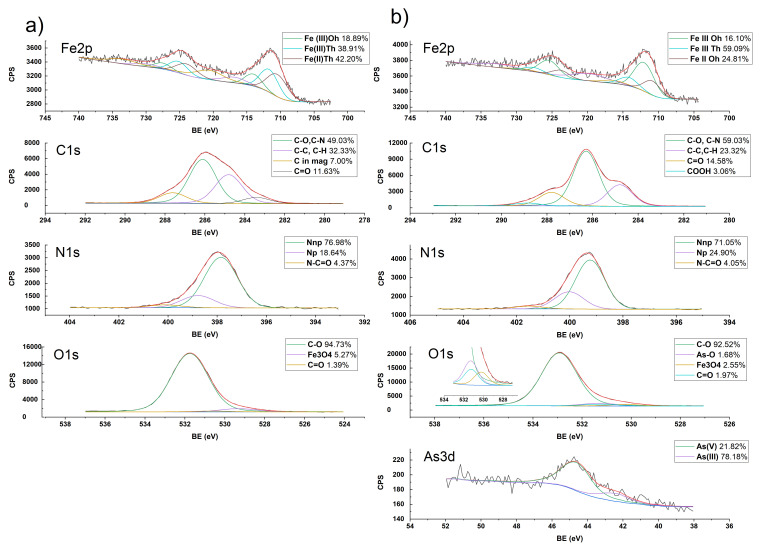
XPS spectra of (**a**) ChM and (**b**) ChM-arsenic.

The XPS data retrieved for the ChM composite, before and after the adsorption of arsenic, is presented in Figure 3. High resolution spectra of ChM were obtained, where displacement correction was performed based on the chitosan signal for the C–C/C–H bonds, assigning them a value of 284.8 eV. In the C1s region, four main components were observed after the deconvolution. The signal at 287.6 eV was assigned to the C=O bonds of the acetylated portion of chitosan (Figure 3a). The signal at 286.1 eV was assigned to C–O and C–N, while the signal at 284.8 eV corresponded to C–C and C–H bonds [34,35,36]. The fourth signal at 283.4 eV is attributed to signals of carbon adsorbed on magnetite [37].

In the deconvolutions of the N1s region, signals from three types of bonds appear. The first signal at 400.1 eV corresponds to the amide group present in the acetylated portions of chitosan. The signals at 399.0 and 397.8 eV are assigned to the amine group in chitosan, with the highest value assigned to the protonated form [34]. It is observed that there is a difference between the measured energy values and the reported values [34,35] for N1s, which can be attributed to the presence of magnetite, which exerts a donor effect causing the decrease of the energy required for the emission of the electrons from the nuclear levels of the nitrogen atoms.

The O1s region (Figure 3a) shows three signals. The most significant signal, at 531.7 eV, is assigned to the C–O bond, the signal at 530.7 eV corresponds to C=O bonds of chitosan [34,35,36], and is minimal (1.39% of the O1s peak area), while the signal at 529.3 eV corresponds to the oxygen atoms of magnetite [37,38,39], and presents 5.27% of the O1s peak area.

In the spectral window of Fe2p (Figure 3a), three main signals, corresponding to Fe(II) and Fe(III) iron ions occupying the tetrahedral (Th) and octahedral (Oh) interstices of magnetite structure, are observed [37]. These signals are consistent with those reported in literature [37]. The signals for Fe(II)_Th_, Fe(III)_Th_ and Fe(III)_Oh_ were assigned at 710.4, 711.6 and 714.0 eV, respectively.

However, the obtained ratio of Fe(II)_Th_:Fe(III)_Th_:Fe(III)_Oh_ is 2:2:1, which differs from the reported ratio of 1:1:1 previously reported for magnetite [37]; this difference is attributed to the interaction of chitosan with iron, increasing the tetrahedral component of iron [37].

When ChM beads were placed in contact with aqueous As(V) solutions, new signals and shifts in some signals were observed because of the interactions between de ChM sorbent and the adsorbed arsenic.

After the adsorption process, the C1s (Figure 3b) signals are maintained at the same binding energies as those observed for ChM (Figure 3a); however, a new signal was observed at 288.9 eV, which is due to the protonation of the carbonyl group of chitosan and residual C compounds in the magnetite. Comparing the spectra obtained for ChM before and after arsenic adsorption, Figure 3b shows that there is a shift of the N1s signals of ChM due to the interaction of arsenic with chitosan, becoming more similar to those energy values reported for chitosan [34]. Figure 3b shows that in the O1s region, a shift of the signal assigned to the C–O and C=O bonds occurs, and the intensity of these signals decreases slightly. A new signal is observed at 531.3 eV that can be assigned to the O–As bonds of the arsenic oxyanions [39].

When arsenate is adsorbed on ChM, the iron signals appear with the same binding energies, but the Fe(II)_Th_:Fe(III)_Th_:Fe(III)_Oh_ ratio changes to 1.3:3.7:1. The increase in Fe(III)Th ratio and decrease in Fe(II) ratio indicates the oxidation of Fe(II)_Th_ to Fe(III)_Th_ [39,40]. The broadening of the curve confirms the oxidation process since the FWHM signal of Fe2p3/2 increases from 4.66 eV to 5.02 eV after adsorption.

The As3d region shows two main signals at 44.37 and 42.41 eV that can be attributed to As(V) and As(III), respectively (Figure 3b). The presence of As(III) corresponds with the transition of Fe(II)_Th_ to Fe(III)_Oh_ observed in the ChM-As complex after adsorption. This electron transfer in which As(V) acts as an electron acceptor, was reported previously [39,40], although the same reports indicate that the reverse process is possible; however, this was not observed in the present experiments.

The modification of the signal intensities and their shifts after arsenic adsorption suggest that magnetite forms a complex with chitosan via nitrogen atoms. The shift in the N1s region is at lower binding energies than previously reported by other authors [34] and the increase in binding energies after adsorption, as well as the subsequent oxidation of iron, support this proposed magnetite–chitosan interaction. Furthermore, this evidence suggests that the binding in the complex is primarily to Fe(II) because, after adsorption, the Fe(II)_Th_:Fe(III)_Th_:Fe(III)_Oh_ ratio drops from 2:2:1 to 1.3:3.7:1. In addition, a decrease in the iron total account is observed; before the adsorption of arsenic, the N:Fe proportion was established in 5.69:1, and after the adsorption of arsenic, the N:Fe proportion was equal to 8.67:1.

### 2.2. Adsorption Kinetics

Table 1 shows the fitting of kinetic data to pseudo-first order model (Equation (4)), pseudo-second order model (Equation (5)) and Elovich model (Equation (6)), as well as the estimated model parameters. Since adsorption models are nonlinear, a nonlinear data regression procedure for the determination of model parameters was applied to avoid the possible distortion of experimental errors produced from a linear analysis [41,42]. Linearization of nonlinear Equations alter the error structure and may disregard the error variance and normality assumptions in the least-squares standard error estimation; nonlinear regression analysis is reported to be more robust [43,44]. The pseudo-first order model yielded the highest correlation coefficients and the lower mean absolute error values (MAE), indicating that it predicts better than the pseudo-second order model and the Elovich model.

**Figure 4:**
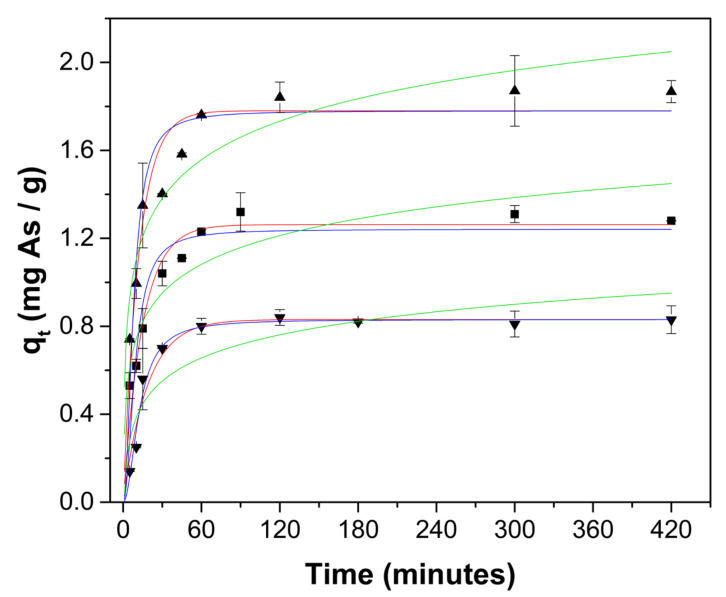
Experimental kinetic data of As adsorption onto ChM hydrogel beads at 25 °C and initial pH = 7.0; using different initial concentrations: 5 mg As/L (▼), 10 mg As/L (■) and (▲) 15 mg As/L. Uptake values predicted by pseudo-first order (—), pseudo-second order (—) and Elovich (—) models.

**Figure 5:**
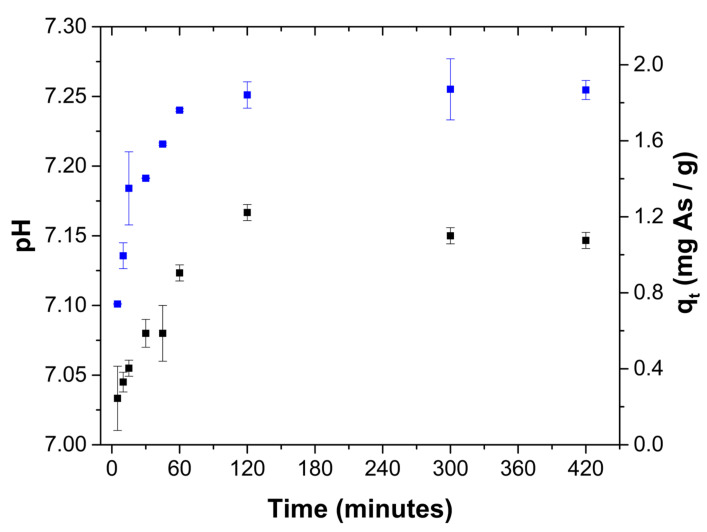
Uptake capacity (■) and pH (■) behavior as a function of contact time between ChM hydrogel beads and arsenate solution at an initial solution pH of 7.0 and an initial concentration of 15 mg As(V)/L.

Figure 4 shows the experimental data and the predicted behavior by the pseudo-first order, pseudo-second order and Elovich models, where it is confirmed that the pseudo-first order model fits the experimental data better. During the adsorption process, the pH of the solution increased with increasing contact time (Figure 5) due to the protonation of mostly amine groups, which are responsible for the arsenate adsorption as suggested by XPS spectra.

### 2.3. Adsorption at Equilibrium

**Figure 6:**
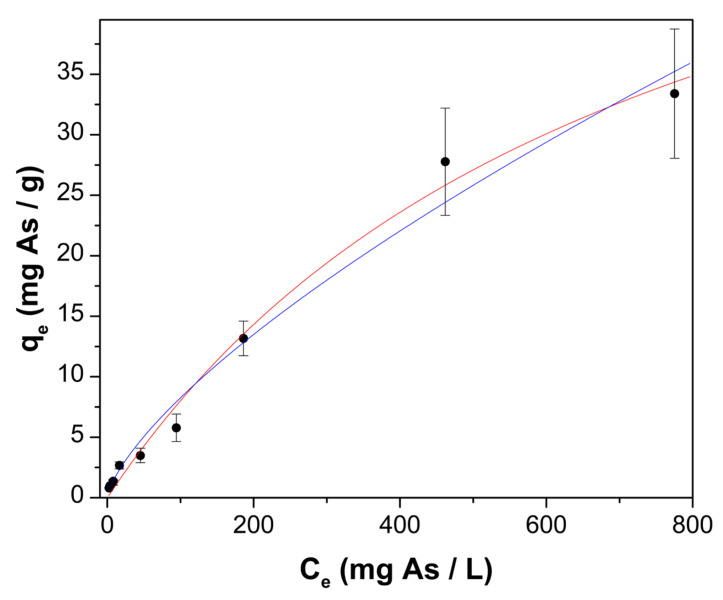
Arsenic adsorption onto ChM at 25 °C and initial pH of 7.0; experimental adsorption isotherm (λ) and predicted data by Langmuir model (—) and Freundlich model (—).

The adsorption isotherm (Figure 6) is L-shaped, without a strict plateau [45]. The isotherm experimental data were fitted by nonlinear regression analysis to the Langmuir (Equation (7)) and Freundlich (Equation (8)) models, since these models are adequate for the analysis of type L isotherms [45]. Table 2 reports the estimated parameters for both models, where higher correlation coefficient values, and lower MAE values, indicate that the Langmuir model best describes the obtained adsorption data. A maximum arsenic uptake capacity of 66.89 ± 8.57 mg As/g was estimated when the initial pH was 7.0.

### 2.4. Effect of pH

Table 3 and Table 4 show C_e_ and q_e_ at an initial solution pH of 6.5 and 7.0, respectively. The sorbent adsorption capacity q_e_ is expressed in milligrams of As per gram of dry sorbent. Table 3 and Table 4 also report that the pH values in liquid phase at the end of the adsorption process are higher than the initial ones. This pH increase is due to the transfer of protons from the solution to the amino groups on the sorbent [46,47]. The pH has an important role in the adsorption mechanism [48].

Higher q_e_ values were obtained when using an initial pH of 6.5 (compared to values with an initial pH = 7.0), which is consistent when taking into consideration that protonated amine groups interact with arsenate species. Particularly, when the initial pH was set at 6.50 ± 0.05, equilibrium was stablished with an increase in pH to up to 6.87–7.07 (Table 4). The difference in pH is important because it defines the proportion of protonated amine groups in the ChM hydrogel beads. The percentage of protonated amine groups of the ChM material was obtained using the Henderson–Hasselbalch equation (Equation (9)), obtaining a percentage of 32.88% protonated amines when the solution of the pH is equal to 6.50. When pH increments to 7.00, the percentage of protonated amines decreases to 13.41%, which is a significant amount that is relevant considering protonated amines are involved in the adsorption of arsenate anions.

### 2.5. Adsorption in a Fixed-Bed

**Figure 7:**
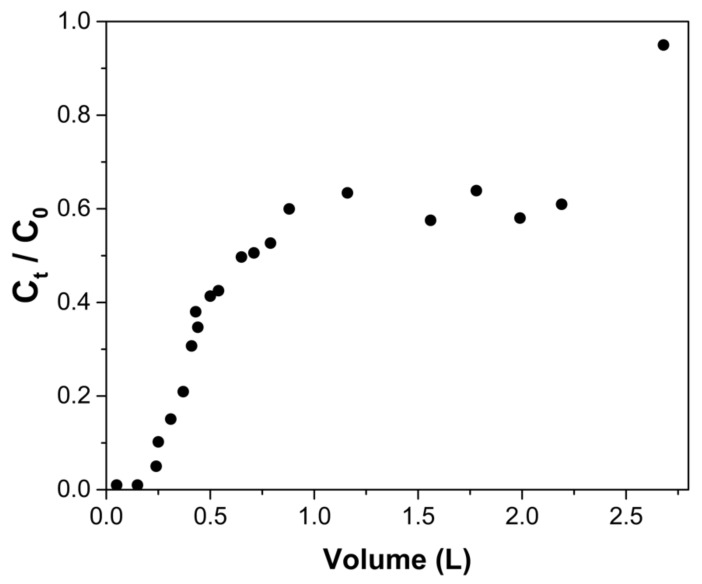
Breakthrough curve for arsenic adsorption using a 13 cm-high column packed with ChM hydrogel beads. Arsenate solution was fed at a rate of 8.5 mL/h, having an initial pH of 7.0 and an initial concentration of 5 mg As(V)/L.

Figure 7 shows that the breakthrough curve has a sigmoidal shape. However, after a volume of approximately 2.3 L has passed through the column, the C_t_/C_0_ ratio increases. The column is considered to reach exhaustion point when a volume of 2.68 ± 0.02 L had passed. Table 5 shows the breakthrough point, which was set when C_t_/C_0_ = 0.05. The number of interstitial bed volumes (NIVB_b_) passed through the column was 28.2 ± 0.2 and the amount of arsenic adsorbed by the ChM was 1.05 ± 0.01 mg As/g. At exhaustion, (NIVB_e_) was equal to 315.4 ± 2.8, and the capacity of the column at this point was 5.68 ± 0.10 mg As/g.

**Figure 8:**
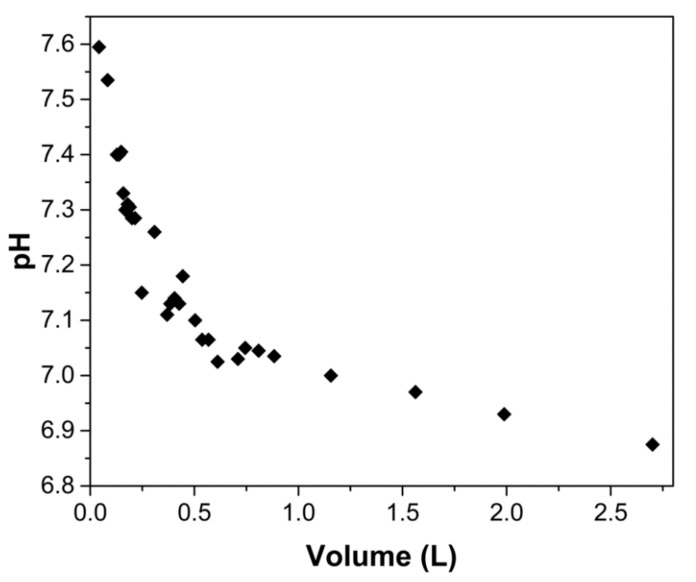
pH of the effluent solution using a 13 cm-high column packed with ChM hydrogel beads. Arsenate solution was fed at a rate of 8.5 mL/h, having an initial pH of 7.0 and a concentration of 5 mg As(V)/L.

Figure 8 shows the pH of the effluent as it exited the column, where it can be observed that at the beginning of the operation, pH increased rapidly to 7.60 ± 0.04. Throughout the column operation, the pH of the effluent slowly decreased, and at the end of the experiment, the pH was 6.88 ± 0.01. The pH increase is due to the transfer of protons from the solution to the chitosan amine groups. Because of the proton transfer from the incoming solution to the amino groups in ChM, new active sites are continuously generated, which favors the adsorption of arsenic. The effluent pH decreases as the solution enters the column because there are fewer amine groups to protonate. When 2.7 L has passed through the column, protonation is almost non-existent, so the effluent concentration increases rapidly, and the column is exhausted.

Curves similar to the one obtained in the present work have been reported [49,50]; after breakthrough occurred, the outflow concentration increased rapidly, then stabilized for a while, then increased again.

The length of the used bed at breakthrough point (H_L_) was estimated to be 2.40 ± 0.10 cm; thus, the length of the unused bed (H_LUB_) was 10.60 ± 0.10 cm, using Equations (12) and (13), respectively. Because the ratio H_LUB_/H_L_ = 4.46, a value that is greater than 1, it is concluded that the mass transfer zone was not fully developed, and the process was not efficient.

## 3. Conclusions

Arsenate adsorption onto ChM hydrogel beads was studied at near-neutral pH, reporting an increase in solution pH as the adsorption of arsenate occurred, which is attributed to the proton transfer to amine groups present in ChM. Despite the complexity of the adsorption process, pseudo-first order was found to best model kinetic data, while Langmuir’s model best described the equilibrium data obtained.

XPS spectra show that arsenate is adsorbed on nitrogen from chitosan and oxygen from magnetite, and that an oxidation of Fe(II) to Fe(III) and a reduction of As(V) to As(III) takes place when de ChM-As complex is formed.

When the As(V) solution with a concentration of 5 mg/L was treated in the fixed-bed column, an amount equal to 28 volumes of the interstitial bed was purified. However, the column was still inefficient because most of the bed was not utilized. To make the process more efficient, it is necessary to have an H_LUB_/H_L_ ratio ≤ 1, which can be accomplished by decreasing the flow rate or increasing the length of the column.

The use of chitosan derivatives as sorbents should imply monitoring pH solutions to better understand and control the process. Solution pH should not be overlooked, as evidenced in the adsorption using a fixed-bed column packed with the ChM hydrogel, where the increments in pH (attributed to proton transfer from the solution to the ChM) lead to a nonideal behavior.

## 4. Materials and Methods

### 4.1. Materials

Food-grade chitosan with a 90% degree of deacetylation was purchased from América Alimentos (Zapopan, México). Magnetite was purchased as Fe_3_O_4_ nanopowder with 50–100 nm particle size (determined by the manufacturer by SEM) from Sigma-Aldrich (Guangzhou, China) and was used as received. Acetic acid and sodium hydroxide were obtained from Fermont (Monterrey, México). Ammonium molybdate tetrahydrate, ascorbic acid, hydrochloric acid and sulphuric acid were purchased from Golden Bell (Zapopan, Mexico). Potassium antimony(III) tartrate hydrate was purchased from Sigma-Aldrich (Bangalore, India). Dibasic sodium arsenate heptahydrate was obtained from Merck (Darmstadt, Germany).

### 4.2. Preparation of ChM Hydrogel Beads

The chitosan powder was first pulverized in a mortar and passed through a 200 mesh sieve. A mass of 4.5 g of powdered chitosan was then dissolved in 100 mL of 2% (*v*/*v*) CH_3_COOH solution. Then, a mass of 0.45 g of magnetite nanopowder was added under stirring to the chitosan solution using a hand-held processor until a dispersion of homogeneous appearance was obtained. For the formation of the ChM hydrogel beads, the dispersion was added dropwise to a 1 M NaOH aqueous solution, using a Masterflex 075557 peristaltic pump with a Masterflex L/S 14 silicone hose. ChM beads were kept for 24 h in the NaOH solution for maturation. Next, the hydrogel beads were washed with bi-distilled water until the obtention of pH = 7.0 in the filtrate. ChM beads were stored in bi-distilled water in refrigeration until used.

### 4.3. Characterization of ChM Hydrogel Beads

#### 4.3.1. Hydrogel Bead Composition

The water content of the hydrogel was obtained by gravimetric analysis; 1.0 g of beads were dried in a MMM Venticell stove at 50 °C until constant weight (Equation (1)). Five replicas were performed for this procedure. Magnetite content of a dry sample was determined by thermogravimetric analysis using a Discovery Thermogravimetric Analyzer (TA Instruments), heating from 25 °C to 600 °C at a rate of 10 °C per minute. The equipment thermobalance’s sensitivity is 0.1 µg. A pulverized ChM sample of 2.2 mg was placed in a platinum sample pan and the mass loss was calculated using the TA Universal Analysis software. Since the magnetite content determined by thermogravimetric analysis corresponds to the dry sample, hydrogel magnetite content was calculated by Equation (2). Chitosan content in the ChM hydrogel was obtained by Equation (3).

(1) $$\%Water=\frac{mass\;of\;hydrogel-dry\;mass}{mass\;of\;hydrogel}100\%$$

(2) $$\%Magnetite_{hydrogel}=\%Magnetite_{dry\;mass}\frac{dry\;mass}{mass\;of\;hydrogel}$$

(3) $$\%Chitosan_{hydrogel}=100\%-\%Water-\%Magnetite_{hydrogel}$$

#### 4.3.2. Average Diameter of Hydrogel Beads

The average diameter of the hydrogel beads was obtained by measuring a sample of 60 beads using a digital electronic calibrator.

#### 4.3.3. Morphology and Surface Characterization of the Hydrogel Beads

The morphology and surface of the ChM hydrogel beads were observed on a Hitachi TM 1000 scanning electron microscope operated at an acceleration voltage of 15.0 kV and an emission current of 48 mA.

#### 4.3.4. Potentiometric Titration of ChM

The pK_a_ of ChM was determined by potentiometric titration following the method reported by Ríos–Donato et al. [33]; a mass of 0.2 g of pulverized ChM was suspended in 10 M HCl and titrated with 0.1 M NaOH. An Ohaus Starter 2100 potentiometer was used for the measurement of pH.

#### 4.3.5. Characterization of Chitosan-Magnetite and ChM-Arsenate Interactions

The interactions between chitosan and magnetite in the ChM composite, and ChM-arsenic were characterized by X-ray Photoelectron Spectroctopy (XPS) using a XPS SPECS System (Berlin, Germany), which contains a Phoibos 150 analyzer and a 1D DLD detector. The XPS spectra were obtained with a monochromatic Al Kα source (1486.6 eV) working at 250 W (12.5 kV and 20 mA) and a base pressure of 3 × 10^−9^ mbar in the analytical chamber. The high-resolution scans were conducted with a pass energy of 15 eV and step sizes of 0.1 eV, using a flood gun source of 20 µA of emission and 2 eV energy to compensate. Data was analyzed with the Analyzer 2.21 software, using Lorentzian–Gaussian curves after background subtraction.

### 4.4. Adsorption Kinetics Studies

Aqueous solutions with different arsenic concentrations (5, 10, and 15 mg As/L) were prepared using Na_2_HAsO_4_·7H_2_O salt and bi-distilled water; the pH of the solution was adjusted to 7.00 ± 0.05 using 0.1 M HCl. A mass of 0.25 g of ChM hydrogel beads was transferred to a 15 mL centrifuge propylene tube, as well as a 10 mL volume of arsenate solution. The tubes were placed in a Thermoshaker (MCR, Accesolab) at 25.0 ± 0.5 °C under continuous agitation (100 RPM). The solutions were separated by decantation at different contact times (5 to 900 min). All adsorption assays were performed in triplicate. The amount of As(V) remaining in the solution was determined using the molybdenum blue method [51]. Solution pH of the samples was measured using an Ohaus 2100 Starter potentiometer.

Kinetic data fitting to mathematical models was carried out by non-linear regression analysis at a 95% significance level, using Statgraphics Centurion XVI software. The Marquardt algorithm was applied for the data analysis. The studied models were pseudo-first order (Equation (4)), pseudo-second order (Equation (5)) and Elovich model (Equation (6)):

(4) $$q_{t}=q_{e}\left(1-e^{-k_{1}t}\right)$$

(5) qt=qek2tk2t+1t

(6) $$q_{t}=\frac{1}{\beta }\ln\left(\alpha \beta \right)\;+\frac{1}{\beta }\ln\left(t\right)$$

where q_t_ is the adsorption capacity at time t, k_1_ and k_2_ are the kinetic constants for the pseudo-first order and pseudo-second order models, respectively. α is the desorption constant and β is the initial adsorption rate [52,53,54].

### 4.5. Adsorption at Equilibrium

Aqueous solutions with different arsenic concentrations, from 4 to 800 mg As/L, were prepared using Na_2_HAsO_4_·7H_2_O salt and bi-distilled water; the pH of the solution was adjusted to 7.00 ± 0.05 using 0.1 M HCl. A mass of 0.25 g of ChM hydrogel beads was transferred to a 15 mL centrifuge propylene tube, as well as a 10 mL volume of arsenate solution. The tubes were placed in a Thermoshaker (MCR, Accesolab) at 25.0 ± 0.5 °C under continuous agitation (100 RPM) for 36 h. Then, the solution was separated from the hydrogel beads by decantation. All treatments were performed in triplicate. The amount of As(V) remaining in the solution was determined using the molybdenum blue method [51]. Solution pH of the samples was measured using an Ohaus 2100 Starter potentiometer.

The experimental isotherm data were fitted to the Langmuir (Equation (7)) and Freundlich (Equation (8)) models by nonlinear regression analysis using the Marquardt algorithm; the analysis was performed using Statgraphics Centurion software.

(7) $$q_{e}=\frac{q_{\max}k_{L}C_{e}}{1+k_{L}C_{e}}$$

(8) qe=kFCe1n

where C_e_ is the solute concentration in the liquid phase at equilibrium, q_e_ is the adsorption capacity at equilibrium, q_m_ is the predicted maximum adsorption capacity, k_L_ is the Langmuir model constant, k_F_ is the Freundlich model constant, and 1/n represents the heterogeneity factor [52,55,56].

### 4.6. Effect of pH

Aqueous solutions with different arsenic concentrations, from 4 to 200 mg As/L, were prepared using Na_2_HAsO_4_·7H_2_O salt and bi-distilled water; the pH of the solution was adjusted to 7.00 ± 0.05 or 6.50 ± 0.005 using 0.1 M HCl. A mass of 0.25 g of ChM hydrogel beads was transferred to a 15 mL centrifuge propylene tube, as well as a 10 mL volume of arsenate solution. The tubes were placed in a Thermoshaker (MCR, Accesolab) at 25.0 ± 0.5 °C under continuous agitation (100 RPM) for 36 h. Then, the solution was separated from the hydrogel beads by decantation. All treatments were performed in triplicate. The amount of As(V) remaining in the solution was determined using the molybdenum blue method [51]. Solution pH of the samples was measured using an Ohaus 2100 Starter potentiometer.

The portion of protonated and unprotonated amine groups of ChM at a given pH value was obtained by the Henderson–Hasselbach equation (Equation (9)), using the pK_a_ value estimated by potentiometric titration.

(9) $$\mathrm{pH}={\mathrm{pK}}_{a}+\log\frac{\left[{\mathrm{NH}}_{2}\right]}{\left[{\mathrm{NH}}_{3}^{+}\right]}$$

### 4.7. Adsorption in a Fixed-Bed

A glass column with an internal diameter of 1.8 cm and a height of 13 cm was packed with 17.2 g of ChM hydrogel beads. An aqueous arsenate solution with an initial concentration of 5 mg As/L was prepared using Na_2_HAsO_4_·7H_2_O salt and bi-distilled water; the pH of the solution was adjusted to 7.00 ± 0.05 using 0.1 M HCl. The column was fed with the arsenate solution using a Masterflex 07557 peristaltic pump with Masterflex L/S 13 silicon hoses, at a flow rate of 8.5 mL/h. The flow direction was from the bottom to the top of the column to avoid channeling of the influent solution [57,58]. Effluent samples were collected at different time intervals. The amount of As(V) remaining in the solution was determined using the molybdenum blue method [51]. Solution pH of the samples was measured using an Ohaus 2100 Starter potentiometer.

Column adsorption capacity values were obtained from the profile of the advancing concentration in the effluent solution at the fixed-bed’s outlet. This profile is referred to as the breakthrough curve and is commonly expressed as the dimensionless concentration C_t_/C_0_ as a function of time or volume. C_t_ is the As(V) concentration of the effluent at time t, while C_0_ is the concentration of the feed solution. Equation (10) is used to estimate column capacity at breakthrough point (q_b_) and Equation (11) is used for the estimation of column capacity at exhaustion (q_e_). Time t_b_ is referred to as the breakthrough time, and it is attained when the concentration of the effluent C_t_ reaches a maximum desired percentage of the feed solution, and its value is usually taken as 1 to 5% of C_0_ [59,60,61]; for the present work, t_b_ was assigned when C_t_ reached 5% of C_0_. Column exhaustion capacity was determined when C_t_ reached 95% of C_0_. In Equations (10) and (11), Q represents the volumetric flow rate and m_ChM_ is the dry mass of ChM hydrogel beads.

(10) $$q_{b}=\frac{{\mathrm{QC}}_{0}}{m_{\mathrm{ChM}}}\int_{0}^{t_{b}}\left(1-\frac{C_{t}}{C_{0}}\right)\mathrm{dt}$$

(11) $$q_{e}=\frac{{\mathrm{QC}}_{0}}{m_{\mathrm{ChM}}}\int_{0}^{t_{e}}\left(1-\frac{C_{t}}{C_{0}}\right)\mathrm{dt}$$

Although, in common practice, a fixed-bed column is stopped once the breakthrough point is reached, it is necessary to continue operating the column until the exhaustion point to be able to determine the length of the used column (H_L_) (Equation (12)) [52,59,62,63]. The ratio q_b_/q_e_ is the fraction of the capacity of the bed used up at the breakthrough point and H represents the total length of the column. H_L_ is the length of the bed used up to the breaking point, this being the region of the column where adsorbent particles are saturated. Therefore, there is a fraction of the column that is not saturated when reaching the breakthrough point, even though it is eventually saturated at exhaustion point, and this fraction of the column is referred to as the length of unused bed (H_LUB_) (Equation (13)), which represents the mass transfer zone (MTZ) developed in the fixed-bed [52,62,63]. The efficiency of the column was referred to as the ration H_LUB_/H_L_.

(12) $$H_{L}=H\frac{q_{b}}{q_{e}}$$

(13) $$H_{\mathrm{LUB}}=H\left(1-\frac{q_{b}}{q_{e}}\right)$$

## Figures and Tables

**Table 1:** Kinetic data of As adsorption by ChM hydrogel beads at 25 °C and initial pH = 7.0, using different initial concentrations of arsenic. The adsorption capacity is expressed in milligrams of As per gram of dry sorbent.

Kinetic Models	C_0_ = 5 mg As/L	C_0_ = 10 mg As/L	C_0_ = 15 mg As/L
Experimental q_e_ (mg As/g)	0.98 ± 0.06	1.36 ± 0.07	1.93 ± 0.14
Pseudo-first order			
Estimated q_e_ (mg As/g)	0.83 ± 0.03	1.26 ± 0.04	1.78 ± 0.06
k_1_ (1/min)	0.055 ± 0.007	0.069 ± 0.008	0.084 ± 0.012
R^2^	0.9582	0.9380	0.9082
MAE	0.036	0.054	0.120
Pseudo-second order			
Estimated q_e_ (mg As/g)	0.83 ± 0.02	1.24 ± 0.05	1.78 ± 0.06
k_2_ (g/mg min)	0.006 ± 0.001	0.012 ± 0.004	0.016 ± 0.004
R^2^	0.9794	0.8568	0.8707
MAE	0.025	0.089	0.096
Elovich			
α	0.192 ± 0.134	0.972 ± 0.751	2.01 ± 1.50
Β	6.60 ± 1.30	5.30 ± 0.88	3.96 ± 0.58
R^2^	0.7871	0.8391	0.8697
MAE	0.108	0.107	0.127

**Table 2:** Langmuir and Freundlich model constants and their correlation coefficients and MAE in As adsorption on ChM hydrogel beads at 25 °C and initial pH = 7.0. Uptake capacity is expressed in As milligrams per gram of dry sorbent.

Langmuir Model	
q_max_ (mg As/g)	66.89 ± 8.57
K_L_ (L/mg As)	0.0014 ± 0.0003
R^2^	0.9913
MAE	0.910
**Freundlich Model**	
N	1.412 ± 0.117
k_F_ (L/g)	0.3165 ± 0.1131
R^2^	0.9837
MAE	0.977

**Table 3:** Arsenic adsorption onto ChM hydrogel beads at equilibrium at 25 °C and initial pH = 7.0.

C_e_ (mg As/L)	q_e_ (mg As/g)	pH_initial_	pH_e_
2.60 ± 0.05	0.81 ± 0.02	6.98	7.22 ± 0.02
3.68 ± 0.10	0.98 ± 0.06	6.99	7.24 ± 0.01
7.92 ± 0.38	1.27 ± 0.23	7.02	7.27 ± 0.01
8.13 ± 0.10	1.36 ± 0.07	6.98	7.20 ± 0.01
16.51 ± 0.48	2.67 ± 0.29	6.98	7.24 ± 0.01
45.29 ± 0.95	3.49 ± 0.60	7.01	7.29 ± 0.01
94.37 ± 1.90	5.78 ± 1.14	6.99	7.25 ± 0.01
186.21 ± 2.19	13.17 ± 1.43	7.04	7.25 ± 0.01

**Table 4:** Arsenic adsorption onto ChM hydrogel beads at equilibrium at 25 °C and initial pH = 6.5.

C_e_ (mg As/L)	q_e_ (mg As/g)	pH_initial_	pH_e_
3.38 ± 0.23	1.25 ± 0.14	6.54	7.03 ± 0.02
7.06 ± 0.13	2.48 ± 0.09	6.55	7.06 ± 0.01
16.06 ± 0.45	3.09 ± 0.15	6.55	7.02 ± 0.02
41.46 ± 0.65	7.64 ± 0.41	6.49	6.95 ± 0.03
85.53 ± 1.71	13.95 ± 1.29	6.52	6.92 ± 0.02
175.53 ± 4.66	26.46 ± 2.16	6.53	6.88 ± 0.01

**Table 5:** Results obtained from the As adsorption at an initial pH of 7.0 using a 13 cm-tall column packed with ChM hydrogel beads. C_0_ = 5 mg/L. Flow rate = 8.5 mL/h.

NIVB_b_	NIVB_e_	q_b_ (mg As/g)	q_e_ (mg As/g)	H_L_ (cm)	H_LUB_ (cm)
2.8 ± 0.2	315.4 ± 2.8	1.05 ± 0.01	5.68 ± 0.10	2.40 ± 0.02	10.60 ± 0.02

## Data Availability

Not applicable.

## References

[1] Fisher A.T., López-Carrillo L., Gamboa-Loira B., Cebrián M.E. (2017). Standards for arsenic in drinking water: Implications for policy in Mexico. J. Public Health Policy.

[2] Flora S.J.S. (2015). Arsenic: Chemistry, Occurrence, and Exposure. Handbook of Arsenic Toxicology.

[3] Kwok K.C.M., Koong L.F., Chen G., McKay G. (2014). Mechanism of arsenic removal using chitosan and nanochitosan. J. Colloid Interface Sci..

[4] Sarkar A., Paul B. (2016). The global menace of arsenic and its conventional remediation—A critical review. Chemosphere.

[5] Kwok K.C.M., Koong L.F., Al Ansari T., McKay G. (2018). Adsorption/desorption of arsenite and arsenate on chitosan and nanochitosan. Environ. Sci. Pollut. Res..

[6] Bujňáková Z., Baláž P., Zorkovská A., Sayagués M.J., Kováč J., Timko M. (2013). Arsenic sorption by nanocrystalline magnetite: An example of environmentally promising interface with geosphere. J. Hazard. Mater..

[7] Oliveira L.H.B., Ferreira N.S., Oliveira A., Nogueira A.R.A., Gonzalez M.H. (2017). Evaluation of distribution and bioaccumulation of arsenic by ICP-MS in tilapia (oreochromis niloticus) cultivated in different environments. J. Braz. Chem. Soc..

[8] Mondal H., Karmakar M., Chattopadhyay P.K., Singha N.R. (2020). Synthesis of pH-responsive sodium alginate-g-tetrapolymers via N–C and O–C coupled in situ monomers: A reusable optimum hydrogel for removal of plant stressors. J. Mol. Liq..

[9] Shahid M., Dumat C., Khalid S., Schreck E., Xiong T., Niazi N.K. (2017). Foliar heavy metal uptake, toxicity and detoxification in plants: A comparison of foliar and root metal uptake. J. Hazard. Mater..

[10] Sekar S., Surianarayanan M., Ranganathan V., MacFarlane D.R., Mandal A.B. (2012). Choline-based ionic liquids-enhanced biodegradation of azo dyes. Environ. Sci. Technol..

[11] Crini G., Badot P.-M. (2008). Application of chitosan, a natural aminopolysaccharide, for dye removal from aqueous solutions by adsorption processes using batch studies: A review of recent literature. Prog. Polym. Sci..

[12] Piccin J.S., Sant’Anna Cadaval T.R.J., Almeida de Pinto L.A., Dotto G.L., Bonilla-Petricolet A., Mendoza-Castillo D.I., Reynel-Ávila H.E. (2017). Adsorption Isotherms in Liquid Phase: Experimental, Modeling and Interpretations. Adsorption Processes for Water Treatment and Purification.

[13] Kurniawan T.A., Chan G.Y.S., Lo W.-H., Babel S. (2006). Comparisons of low-cost adsorbents for treating wastewaters laden with heavy metals. Sci. Total Environ..

[14] Leyva Ramos R., Litter M.I., Sancha A.M., Ingallinella A.M. (2010). Fundamentos de adsorción en sistemas líquido-sólido. Tecnologías Económicas para el Abatimiento de Arsénico en Aguas.

[15] Bhatnagar A., Sillanpää M. (2009). Applications of chitin- and chitosan-derivatives for the detoxification of water and wastewater—A short review. Adv. Colloid Interface Sci..

[16] Gerente C., Lee V.K.C., Le Cloirec P., McKay G. (2007). Application of Chitosan for the Removal of Metals From Wastewaters by Adsorption—Mechanisms and Models Review. Crit. Rev. Environ. Sci. Technol..

[17] Zargar V., Asghari M., Dashti A. (2015). A Review on Chitin and Chitosan Polymers: Structure, Chemistry, Solubility, Derivatives, and Applications. ChemBioEng Rev..

[18] Liu C., Bai R. (2014). Recent advances in chitosan and its derivatives as adsorbents for removal of pollutants from water and wastewater. Curr. Opin. Chem. Eng..

[19] Wan Ngah W.S., Teong L.C., Hanafiah M.A.K.M. (2011). Adsorption of dyes and heavy metal ions by chitosan composites: A review. Carbohydr. Polym..

[20] Rouf S., Nagapadma M. (2015). Modeling of Fixed Bed Column Studies for Adsorption of Azo Dye on Chitosan Impregnated with a Cationic Surfactant. Int. J. Sci. Eng. Res..

[21] Varma A.J., Deshpande S.V., Kennedy J.F. (2004). Metal complexation by chitosan and its derivatives: A review. Carbohydr. Polym..

[22] Gotoh T., Matsushima K., Kikuchi K.I. (2004). Preparation of alginate-chitosan hybrid gel beads and adsorption of divalent metal ions. Chemosphere.

[23] Gang D.D., Deng B., Lin L.S. (2010). As(III) removal using an iron-impregnated chitosan sorbent. J. Hazard. Mater..

[24] Vieira M.L.G., Esquerdo V.M., Nobre L.R., Dotto G.L., Pinto L.A.A. (2014). Glass beads coated with chitosan for the food azo dyes adsorption in a fixed bed column. J. Ind. Eng. Chem..

[25] Singha N.R., Deb M., Chattopadhyay P.K., Rangappa S.M., Parameswaranpillai J., Siengchin S., Ramesh M. (2022). Chitin and chitosan-based blends and composites. Biodegradable Polymers, Blends and Composites.

[26] Chen C.C., Chung Y.C. (2006). Arsenic removal using a biopolymer chitosan sorbent. J. Environ. Sci. Health—Part A Toxic/Hazardous Subst. Environ. Eng..

[27] Carneiro M.A., Pintor A.M.A., Boaventura R.A.R., Botelho C.M.S. (2022). Efficient removal of arsenic from aqueous solution by continuous adsorption onto iron-coated cork granulates. J. Hazard. Mater..

[28] Pontoni L., Fabbricino M. (2012). Use of chitosan and chitosan-derivatives to remove arsenic from aqueous solutions—A mini review. Carbohydr. Res..

[29] Gupta A., Chauhan V.S., Sankararamakrishnan N. (2009). Preparation and evaluation of iron-chitosan composites for removal of As(III) and As(V) from arsenic contaminated real life groundwater. Water Res..

[30] Wang J., Xu W., Chen L., Huang X., Liu J. (2014). Preparation and evaluation of magnetic nanoparticles impregnated chitosan beads for arsenic removal from water. Chem. Eng. J..

[31] Ayub A., Raza Z.A., Majeed M.I., Tariq M.R., Irfan A. (2020). Development of sustainable magnetic chitosan biosorbent beads for kinetic remediation of arsenic contaminated water. Int. J. Biol. Macromol..

[32] Bolisetty S., Peydayesh M., Mezzenga R. (2019). Sustainable technologies for water purification from heavy metals: Review and analysis. Chem. Soc. Rev..

[33] Ríos Donato N., Navarro R., Avila Rodríguez M., Mendizábal E. (2012). Coagulation–flocculation of colloidal suspensions of kaolinite, bentonite, and alumina by chitosan sulfate. J. Appl. Polym. Sci..

[34] Maachou H., Genet M.J., Aliouche D., Dupont-Gillain C.C., Rouxhet P.G. (2013). XPS analysis of chitosan-hydroxyapatite biomaterials: From elements to compounds. Surf. Interface Anal..

[35] Guerra S., Barbera V., Vitale A., Bongiovanni R., Serafini A., Conzatti L., Brambilla L., Galimberti M. (2020). Edge functionalized graphene layers for (ultra) high exfoliation in carbon papers and aerogels in the presence of chitosan. Materials.

[36] Gieroba B., Sroka-Bartnicka A., Kazimierczak P., Kalisz G., Lewalska-Graczyk A., Vivcharenko V., Nowakowski R., Pieta I.S., Przekora A. (2020). Spectroscopic studies on the temperature-dependent molecular arrangements in hybrid chitosan/1,3-β-D-glucan polymeric matrices. Int. J. Biol. Macromol..

[37] Lesiak B., Rangam N., Jiricek P., Gordeev I., Tóth J., Kövér L., Mohai M., Borowicz P. (2019). Surface Study of Fe3O4 Nanoparticles Functionalized With Biocompatible Adsorbed Molecules. Front. Chem..

[38] Goretzki H., Rosenstiel P.V., Mandziej S. (1989). Standard Reference Data. Fresenius’ Z. Anal. Chem..

[39] Chowdhury S.R., Yanful E.K., Pratt A.R. (2011). Arsenic removal from aqueous solutions by mixed magnetite-maghemite nanoparticles. Environ. Earth Sci..

[40] Liu C.H., Chuang Y.H., Chen T.Y., Tian Y., Li H., Wang M.K., Zhang W. (2015). Mechanism of Arsenic Adsorption on Magnetite Nanoparticles from Water: Thermodynamic and Spectroscopic Studies. Environ. Sci. Technol..

[41] Foo K.Y., Hameed B.H. (2010). Insights into the modeling of adsorption isotherm systems. Chem. Eng. J..

[42] Bonilla-Petricolet A., Mendoza-Castillo D.I., Reynel-Ávila H.E., Bonilla-Petricolet A., Mendoza-Castillo D.I., Reynel-Ávila H.E. (2017). Introduction. Adsorption Processes for Water Treatment and Purification.

[43] Das P., Banerjee P., Rathour R., Misra R. (2015). Assessment on linear and non-linear analysis for the estimation of pseudo-second-order kinetic parameters for removal of dye using graphene nanosheet. Desalin. Water Treat..

[44] González-López M.E., Laureano-Anzaldo C.M., Pérez-Fonseca A.A., Arellano M., Robledo-Ortíz J.R. (2021). A discussion on linear and non-linear forms of Thomas equation for fixed-bed adsorption column modeling. Rev. Mex. Ing. Química.

[45] Limousin G., Gaudet J.P., Charlet L., Szenknect S., Barth??s V., Krimissa M. (2007). Sorption isotherms: A review on physical bases, modeling and measurement. Appl. Geochemistry.

[46] Fourest E., Volesky B. (1996). Contribution of Sulfonate Groups and Alginate to Heavy Metal Biosorption by the Dry Biomass of Sargassum fluitans. Environ. Sci. Technol..

[47] Verduzco-Navarro I.P., Rios-Donato N., Jasso-Gastinel C.F., Martínez-Gómez Á.D.J., Mendizábal E. (2020). Removal of Cu(II) by Fixed-Bed Columns Using Alg-Ch and Alg-ChS Hydrogel Beads: Effect of Operating Conditions on the Mass Transfer Zone. Polymers.

[48] Igberase E., Osifo P., Ofomaja A. (2014). The adsorption of copper (II) ions by polyaniline graft chitosan beads from aqueous solution: Equilibrium, kinetic and desorption studies. J. Environ. Chem. Eng..

[49] Da Luz Mesquita P., Ribeiro Souza C., Santos N.T.G., Ferreira Rocha S.D. (2018). Fixed-bed study for bone char adsorptive removal of refractory organics from electrodialysis concentrate produced by petroleum refinery. Environ. Technol..

[50] Verduzco-Navarro I.P., Jasso-Gastinel C.F., Ríos-Donato N., Mendizábal E. (2020). Red dye 40 removal by fixed-bed columns packed with alginate-chitosan sulfate hydrogels. Rev. Mex. Ing. Química.

[51] Murphy K., Riley J.P. (1962). A modified single solution method for the determination of phosphate in natural waters. Anal. Chim. Acta.

[52] Dotto G.L., Gonçalves Salau N.P., Piccin J.S., Sant’Anna Cadaval T.R.J., Almeida de Pinto L.A., Bonilla-Petricolet A., Mendoza-Castillo D.I., Reynel-Ávila H.E. (2017). Adsorption Kinetics in Liquid Phase: Modeling for Discontinuous and Continuous Systems. Adsorption Processes for Water Treatment and Purification.

[53] Qiu H., Lv L., Pan B., Zhang Q., Zhang W., Zhang Q. (2009). Critical review in adsorption kinetic models. J. Zhejiang Univ. Sci. A.

[54] Ho Y.S., McKay G. (1998). A Comparison of chemisorption kinetic models applied to pollutant removal on various sorbents. Process Saf. Environ. Prot..

[55] Azalea Z., Marsin M., Ibrahim W., Aini W. (2021). Alginate-based adsorbents for removal of metal ions and radionuclides from aqueous solutions: A review. Int. J. Biol. Macromol..

[56] Dada A.O., Olalekan A.P., Olatunya A.M., Dada O.J.I.J.C., Langmuir F. (2012). Temkin and Dubinin—Radushkevich Isotherms Studies of Equilibrium Sorption of Zn 2 + Unto Phosphoric Acid Modified Rice Husk. IOSR J. Appl. Chem..

[57] Boddu V.M., Abburi K., Talbott J.L., Smith E.D., Haasch R. (2008). Removal of arsenic (III) and arsenic (V) from aqueous medium using chitosan-coated biosorbent. Water Res..

[58] Dhoble R.M., Maddigapu P.R., Rayalu S.S., Bhole A.G., Dhoble A.S., Dhoble S.R. (2017). Removal of arsenic(III) from water by magnetic binary oxide particles (MBOP): Experimental studies on fixed bed column. J. Hazard. Mater..

[59] Cussler E.L. (2009). Adsorption. Diffusion.

[60] Geankoplis C.J., Hersel A.A., Lepek D.H. (2018). Transport Processes and Separation Process Principles.

[61] Patel H., Vashi R.T. (2015). Fixed-Bed Column Studies of Dyeing Mill Wastewater Treatment Using Naturally Prepared Adsorbents. Characterization and Treatment of Textile Wastewater.

[62] Barros M.A.S.D., Arroyo P.A., Silva E.A., Nakajima H. (2013). General Aspects of Aqueous Sorption Process in Fixed Beds. Mass Transfer—Advances in Sustainable Energy and Environment Oriented Numerical Modeling.

[63] Kopsidas O. (2016). Scale Up of Adsorption in Fixed-Bed Column Systems.

